# Synergistic Multi-Mechanism Enhancement in Chemomechanical Abrasive Polishing of Polycrystalline Diamond via a New SiO_2_–Diamond Slurry in High-Concentration H_2_O_2_ Solution

**DOI:** 10.3390/ma18153659

**Published:** 2025-08-04

**Authors:** Xin Zheng, Ke Zheng, Jie Gao, Yan Wang, Pengtao An, Yongqiang Ma, Hongjun Hei, Shuaiwu Qu, Shengwang Yu

**Affiliations:** College of Materials Science and Engineering, Taiyuan University of Technology, Taiyuan 030024, China; zhengxin0055@link.tyut.edu.cn (X.Z.); zhengke@tyut.edu.cn (K.Z.); 2024510265@link.tyut.edu.cn (Y.W.); 2024520252@link.tyut.edu.cn (P.A.); 2024520351@link.tyut.edu.cn (Y.M.); heihongjun@tyut.edu.cn (H.H.); 2023310057@link.tyut.edu.cn (S.Q.)

**Keywords:** polycrystalline diamond (PCD), chemomechanical abrasive polishing (CMAP), material removal rate (MRR), surface roughness, SiO_2_–diamond slurry, removal mechanism of diamond material

## Abstract

The high-efficiency polishing of large-sized polycrystalline diamond (PCD) wafers continues to pose significant challenges in its practical applications. Conventional mechanical polishing suffers from a low material removal rate (MRR) and surface damage. To improve the process efficiency, this study investigates the effect of chemomechanical abrasive polishing (CMAP) with a slurry containing high-concentration H_2_O_2_ and varying mass percentages of SiO_2_ powder and diamond particles on surface morphology, surface roughness, material removal rate (MRR), and microstrain of PCD disks. The contributions of mechanical action, chemical action, and bubble cavitation to the CMAP process are analyzed. Scanning electron microscopy (SEM) observations indicate that large grains present in PCD are effectively eliminated after CMAP, leading to a notable reduction in surface roughness. The optimal results are obtained with 60 wt% SiO_2_ powder and 40 wt% diamond particles, achieving a maximum MRR of 1039.78 μm/(MPa·h) (15.5% improvement compared to the mechanical method) and a minimum surface roughness (Sa) of 3.59 μm. Additionally, the microstrain on the PCD disk shows a slight reduction following the CMAP process. The material removal mechanism is primarily attributed to mechanical action (70.8%), with bubble cavitation and chemical action (27.5%) and action of SiO_2_ (1.7%) playing secondary roles. The incorporation of SiO_2_ leads to the formation of a lubricating layer, significantly reducing surface damage and decreasing the surface roughness Sa to 1.39 µm.

## 1. Introduction

Polycrystalline diamond (PCD) has developed rapidly in recent decades owing to its superior properties [1,2,3]. In the field of PCD applications, surface roughness is a critical factor that significantly influences its performance. For instance, a rough surface on polycrystalline diamond (PCD) can significantly reduce heat dissipation efficiency and impair optical transparency due to light scattering [2]. Consequently, the surface polishing of PCD is an essential step to enhance its performance and broaden its applicability. However, the exceptional anisotropy, remarkable mechanical properties, high wear resistance, strong chemical inertness, and heterogeneity of diamond make its high-efficiency and high-precision processing a formidable challenge [4].

Conventional diamond polishing typically employs a mechanical polishing method, which not only exhibits a low material removal rate (MRR) but also is prone to inducing significant surface and subsurface damage. In 1975, Thornton and Wilks presented the concept of chemical mechanical polishing (CMP) for diamond [5], which can almost avoid damage through the synergies between chemical and mechanical interactions. H.Y. Tsai [6] compared mechanical polishing and CMP, and the result showed that the surface roughness of PCD polished by CMP was much lower than that obtained with mechanical polishing. Thomas [7] realized a lower surface roughness after polishing with a SiO_2_-based alkaline colloidal slurry (Logitech SF1 Syton). The surface roughness value was reduced from 0.92 to 0.23 nm and from 0.31 to 0.09 nm rms for {100} and {111} single-crystal diamond, respectively. Jessica M. Werrell [8] compared the polishing efficiency of three types of polishing particles—CeO_2_, Al_2_O_3_, and SiO_2_— in the Logitech SF1 Syton slurry, and the results demonstrated that adding SiO_2_ particles led to the highest polishing efficiency. In addition to oxide abrasive particles, a strong oxidizing agent, such as an H_2_O_2_ solution, incorporated into the slurry can significantly enhance the polishing efficiency. Akihisa Kubota [9] improved the MRR of single-crystal diamond by adding an H_2_O_2_ solution on a Ni plate in the polishing process, and the result showed a higher MRR of 216.7 nm/h and a lower surface roughness of 0.1 nm compared to the values obtained without H_2_O_2_. Further, Soumen Mandal [10] showed a ~15% value decrease in the surface roughness of diamond by adding a 20% H_2_O_2_ solution in the Logitech SF1 Syton slurry during the polishing process. These previous studies concluded that both an H_2_O_2_ solution and SiO_2_ particles can improve the MRR and reduce the surface roughness of diamond after polishing. However, in these studies, the concentration of the employed H_2_O_2_ solution was about 20% or lower and the percentage of SiO_2_ was in a certain range. The influence of a varying SiO_2_ content and higher concentrations of the H_2_O_2_ solution on polishing efficiency remains unexplored.

In addition, with the increasing diameter and thickness of PCD wafers, the grain size rises accordingly, thereby substantially elevating the difficulty of surficial processes. Moreover, the polishing process of large PCD wafers always requires a long time, which illustrates the necessity of increasing its efficiency. In general, the polishing process can be divided into two phases: first, the removal of large grains, and finally the poling process. The former phase can be regarded as a pre-treatment. The pre-treatment is needed to remove large diamond grains and reduce the surface roughness as far as possible before the final polishing. To accomplish it, diamond particles are generally employed. In this study, to increase the efficiency of the pre-treatment, chemomechanical abrasive polishing (CMAP) is proposed based on the technic of CMP (improvement in diamond material removal). Different mass percentages of SiO_2_ powder and diamond particles are added to a high-concentration H_2_O_2_ solution to form a CMAP slurry, and their effect on the surface treatment of large PCD wafers is investigated. In addition, the removal mechanisms for PCD are analyzed and discussed quantitatively for the first time. Surface variation for self-deposited PCD wafers during the pre-treatment is also characterized by SEM, EDS, Raman spectroscopy, XRD, and XPS.

## 2. Experimental Details

### 2.1. Materials and Methods

A PCD wafer was deposited on a (100) silicon substrate (diameter of 120 mm) by a self-developed TYUT-type microwave plasma chemical vapor deposition (MPCVD) equipment (915 MHz, 25 kW). The detailed deposition parameters are listed in Table 1. Prior to deposition, the Si substrate was mechanically abraded with a 1000# diamond powder and then ultrasonically cleaned. After deposition for about ~720 h, the PCD wafer reached a thickness of around 1 mm and was sliced into disks with a diameter of 15 mm. Before the CMAP process, each disk underwent thickness measurement procedures at five different locations, conducted with a precision of 1 µm using a calibrated micrometer. The measurements were subsequently averaged to establish a base value for the following comparison.

**Figure 1:**
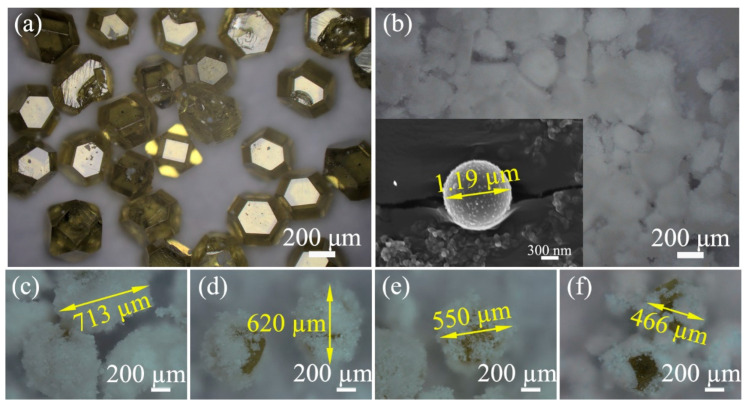
Optical photographs of (**a**) diamond particles; (**b**) SiO_2_ powder; (**c**–**f**) mixtures of abrasive particles, consisting of (**c**) 20 wt% diamond particles and 80 wt% SiO_2_ powder, (**d**) 40 wt% diamond particles and 60 wt% SiO_2_ powder, (**e**) 60 wt% diamond particles and 40 wt% SiO_2_ powder, and (**f**) 80 wt% diamond particles and 20 wt% SiO_2_ powder.

**Figure 2:**
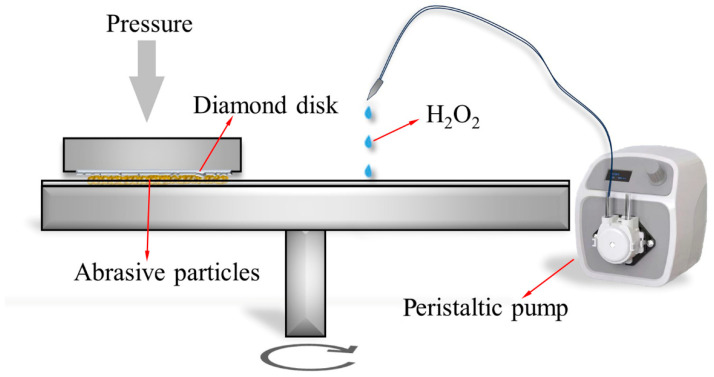
Schematic diagram of the CMAP process.

The experimental materials comprised diamond abrasive particles (50–60 mesh, Borui Henan), a submicron silicon dioxide (SiO_2_) powder with a mean particle size of approximately 1 μm, and a hydrogen peroxide (H_2_O_2_) solution at a standardized concentration of 30% (analytical-grade purity). The mass ratio of SiO_2_ with respect to the abrasive particles was increased from 20 wt% to 80 wt%, and correspondingly, the mass content of the diamond particles decreased sequentially. Figure 1 presents optical photographs characterizing the abrasive particles, i.e., (a) the diamond particles and (b) the SiO_2_ powder. Figure 1c–f displays the composite mixtures of diamond particles and SiO_2_ powder at varying mass percentages. The images reveal a distinct adsorption phenomenon, wherein the SiO_2_ powder exhibits strong surface adherence to the diamond particles, attributed to the different surface energy of the two materials. Furthermore, it can be observed that the exposed area of the diamond particles increases, and the diameter of the diamond particles adsorbing SiO_2_ decreases as the mass percentage of the SiO_2_ powder is reduced from 713 µm to 466 µm. The schematic diagram illustrating the CMAP process is shown in Figure 2. Free abrasive particles with a weight of 30 g were placed between the PCD disk and the metal plate first. Then the CMAP process was conducted under controlled conditions, with a pressure of 0.15 MPa applied on the polycrystalline diamond (PCD) disk and a rotation speed of ~0.4 m/s, for a duration of 1 h. Following the CMAP process, the treated PCD disks underwent ultrasonic cleaning in deionized water to remove residual abrasive particles and polishing byproducts. Their thickness was measured to accurately quantify the material removal rates and height loss. Three samples under the indicated conditions were tested, and the corresponding results were analyzed statistically, calculating their mean values and standard deviation and then visually exhibiting them.

### 2.2. Characterization

The macro-morphology analysis of the deposited Φ120 mm PCD wafer was conducted by a camera. The surface morphology of the PCD disks before and after CMAP was examined by a ZEISS Gemini 300 scanning electron microscope (SEM). The quality of the PCD wafer was evaluated using a Raman spectrometer with a laser excitation wavelength of 532 nm (Renishaw Invial, Wotton-under-Edge, UK). The orientation and microstrain of the PCD disks were characterized using a Rigaku Smartlab X-ray (Tokyo, Japan) diffractometer (XRD). The surface roughness of the PCD disks before and after CMAP was examined by a 3D digital graphical microscope (VHX-7000 970F), in which five locations including one center and four edges were selected. Each measured area was ~1.5 mm^2^. Further, X-ray photoelectron spectroscopy (XPS, Thermo Scientific ESCALAB Xi+, Waltham, MA, USA) was conducted on each PCD disk before and after CMAP to survey the variation in surface bonding. To further analyze the mechanical removal mechanism for PCD, single diamond particles with different crystalline surfaces were brazed on 42CrMo steel (brazing temperature 1293 K, holding time 5 min, furnace pressure < 5 Pa, protection gas Ar) and ground by a diamond wheel (1500 r/min). Then, the stress distribution at the particle/PCD interface during the CMAP process was simulated by COMSOL. For the chemical analysis, changes in surface C element in the diamond particles were investigated after CMAP based on the Raman spectra. Finally, an extra experiment was conducted to identify the contribution of different mechanisms to PCD material removal.

## 3. Experimental Results

**Figure 3:**
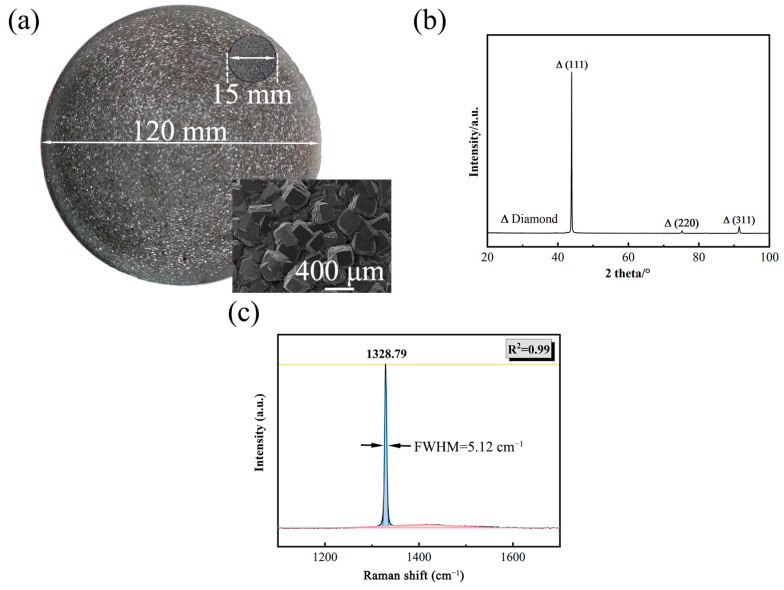
(**a**) Optical photograph and SEM photograph (inset image) showing the morphology of the as-deposited PCD wafer; (**b**) XRD pattern; (**c**) typical Raman spectrum of the PCD wafer.

Figure 3 shows both the macro-morphology and a typical Raman spectrum of the as-deposited PCD wafer. Figure 3a displays a uniform PCD wafer with a diameter of 120 mm, which shows no significant deviations, illustrating a homogeneous diamond growth. The inset image shows the macro-morphology and micro-morphology of the PCD disk with a diameter of 15 mm, which reveals distinct crystal planes. The observed rectangular top surface represents the (100) crystal plane, and the textured side faces correspond to the (111) crystal plane, in which the observed special morphology was mainly caused by a faster growth speed of the (111) crystal plane, resulting in the exposure of the (100) crystal plane on the upper surface [11]. In Figure 3b, the XRD pattern of the PCD wafer is exhibited, in which the peaks at 2θ = 43.9°, 2θ = 75.3°, and 2θ = 91.4° correspond to the e (111), (110), and (311) diamond crystal planes. Among them, the (111) crystal plane is the preferred orientation, which is consistent with the SEM results. The typical Raman spectroscopic analysis of the PCD wafer is presented in Figure 3c, revealing a characteristic first-order diamond phonon peak centered at 1328.79 cm^−1^ and a full width at half maximum (FWHM) of 5.12 cm^−1^. However, a weak broad peak indicated in pink was attributed to the D band of sp^2^ carbon and suggests a medium quality of the deposited PCD wafer. During diamond deposition, C_x_H_y_ groups and free atomic hydrogen from methane and hydrogen plasma [12,13,14] drive the nucleation and growth of diamond and graphite on the Si substrate. At the same time, these phases are also etched by free atomic hydrogen, with graphite being etched more rapidly. If the growth rate exceeds the etching rate, residual amorphous carbon and graphite remain in the diamond film.

**Figure 4:**
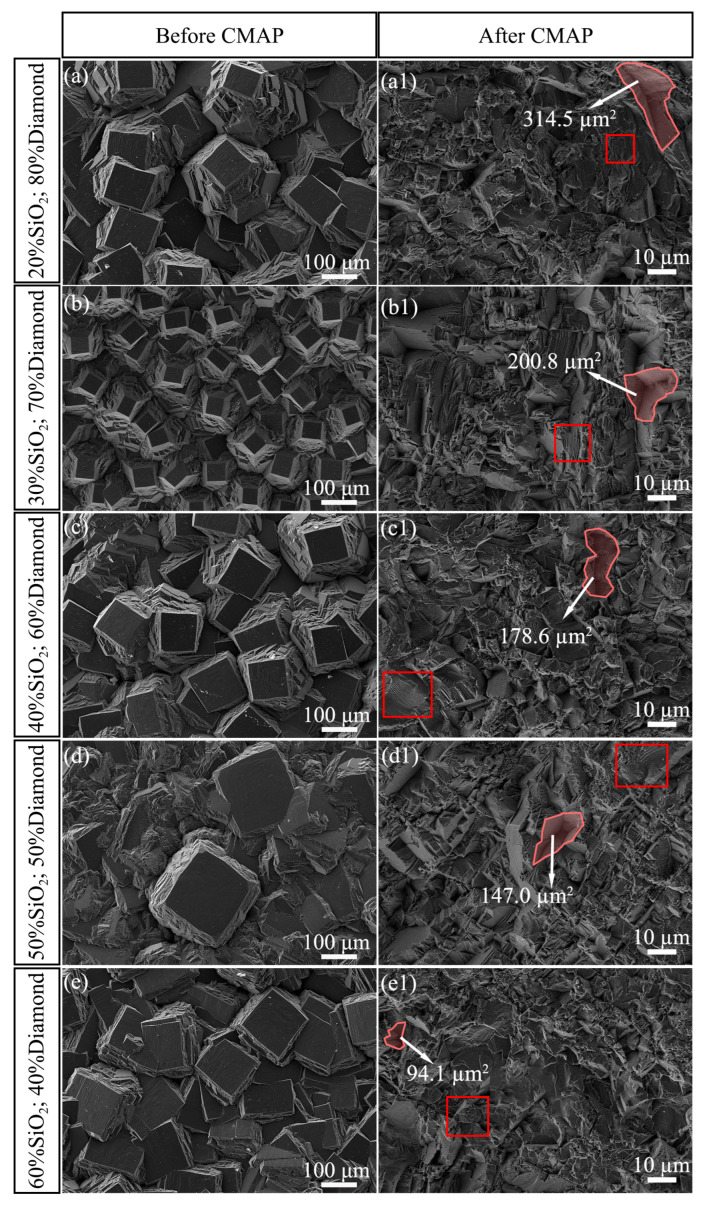

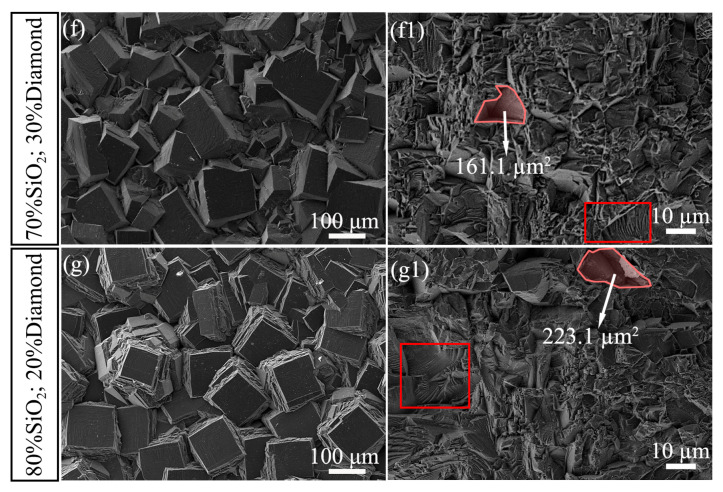
Surface morphology of PCD disks before and after CMAP. Abrasive mixtures consisting of (**a**,**a1**) 20%SiO_2_–80%diamond; (**b**,**b1**) 30%SiO_2_–70%diamond; (**c**,**c1**) 40%SiO_2_–60%diamond; (**d**,**d1**) 50%SiO_2_–50%diamond; (**e**,**e1**) 60%SiO_2_–40%diamond; (**f**,**f1**) 70%SiO_2_–30%diamond; (**g**,**g1**) 80%SiO_2_–20%diamond.

Figure 4 shows the surface morphology of the PCD disks analyzed by SEM before and after the CMAP process. The images in Figure 4a–g demonstrate consistent crystal orientation across all PCD disk surfaces, with the (100) crystal plane predominantly exposed, regardless of the different initial grain sizes. The post-CMAP surface morphologies presented in Figure 4(a1–g1) demonstrate significant changes following the removal of large diamond grains and the elimination of distinct grain boundaries, revealing a substantial reduction in surface roughness. Notably, the post-CMAP surfaces display a complex morphology comprising two distinct features: predominant brittle cleavage fracture patterns of the diamond (marked by red rectangles) and isolated pits with smooth internal surfaces (marked by a pink contour). The distinct surface topography is likely attributable to different crystal orientations and the presence of intrinsic defects, including grain boundaries, dislocations, twins, and vacancies introduced during deposition [15]. Generally, under external mechanical pressure, microcracks first appear along the growth defects and then expand and aggregate. Finally, large diamond fragments peel off from the surface following brittle cleavage fractures [16]. A quantitative analysis of the surface morphology showed that the area of each of the biggest pits observed in the CMAP-treated PCD disks presented in Figure 4(a1–g1) was 314.5 µm^2^, 200.8 µm^2^, 178.6 µm^2^, 147.0 µm^2^, 94.1 µm^2^, 161.1 µm^2^, and 223.1 µm^2^, respectively. The surface area of the biggest pits demonstrated a non-monotonic variation, initially decreasing and then increasing, exhibiting a strong correlation with the reduction in mass percentage of the diamond particles and the concomitant increase in SiO_2_ mass. This phenomenon can be primarily ascribed to the distinct abrasive polishing effects induced by the variation in the mass percentages of diamond and SiO_2_ particles and will be analyzed and discussed in detail below.

**Figure 5:**
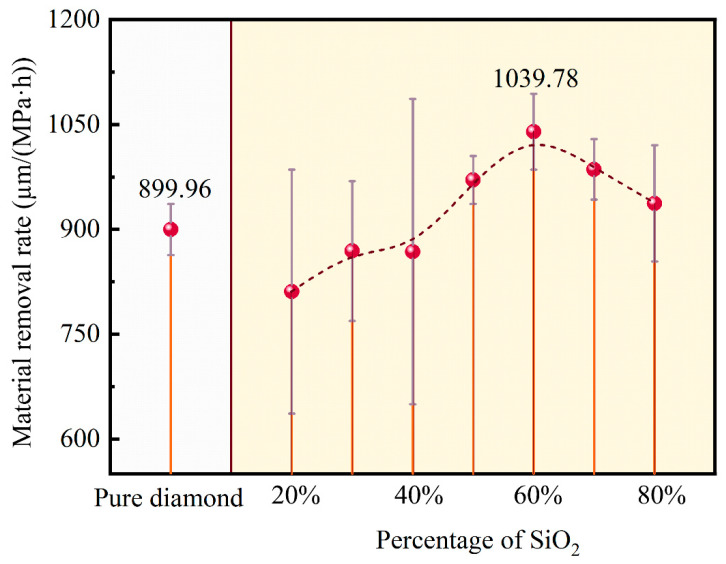
The MRR for the PCD disks.

Figure 5 presents the MRR values for the PCD disks. The reference value of 899.96 µm/(MPa·h), attained through processing with pure diamond particles, was regarded as a baseline for a comparative analysis of the results obtained using the slurries containing varying mass percentages of diamond particles and SiO_2_ powder. As exhibited by the data presented in the right part of Figure 5, the MRR showed a distinct changing trend, characterized by an initial increasing tendency followed by a subsequent decline, correlating directly with the progressive increase in mass percentage of the SiO_2_ powder. The optimal MRR value of 1039.78 µm/(MPa·h) was achieved at 60 wt% SiO_2_, representing a significant enhancement of 15.5% compared to that obtained with pure diamond particles.

This variation in MRR was mainly attributed to changes in stress induced by the contact between the diamond particles and the PCD disk. The small-sized SiO_2_ powder adhering to the surfaces of both diamond particles and PCD disk could change the contact pattern. When the mass percentage of SiO_2_ powder was in a low range, the pressure exerted on the PCD surface did not change significantly, and the spherical SiO_2_ powder particles reduce the direct contact between the diamond particles and the PCD disk, consequently leading to a lower MRR compared to that achieved by abrasion with pure diamond particles. As the mass percentage of SiO_2_ rose, the number of diamond particles involved in the CMAP process correspondingly decreased, thereby increasing the local contact stress and altering the contact configuration to one consisting of PCD disk–SiO_2_–diamond particles. In these conditions, the PCD disk was subjected to higher pressure transmitted by the diamond particles through the adhering SiO_2_ layer; the deformed SiO_2_ powder could also increase the effective contact area with the PCD disk and enable a more even pressure distribution. The even and appropriate pressure increased the efficiency of material removal. In addition, the large specific surface area of SiO_2_ could increase the contact area between H_2_O_2_ and the PCD disk. Hence, the MRR increased when the contact was “soft”. Nevertheless, when the SiO_2_ percentage became excessively high, the quantity of diamond particles participating in the CMAP process decreased significantly. Additionally, due to the difference in physical properties between SiO_2_ and diamond, the material removal efficiency was low, ultimately leading to a decrease in the MRR.

**Figure 6:**
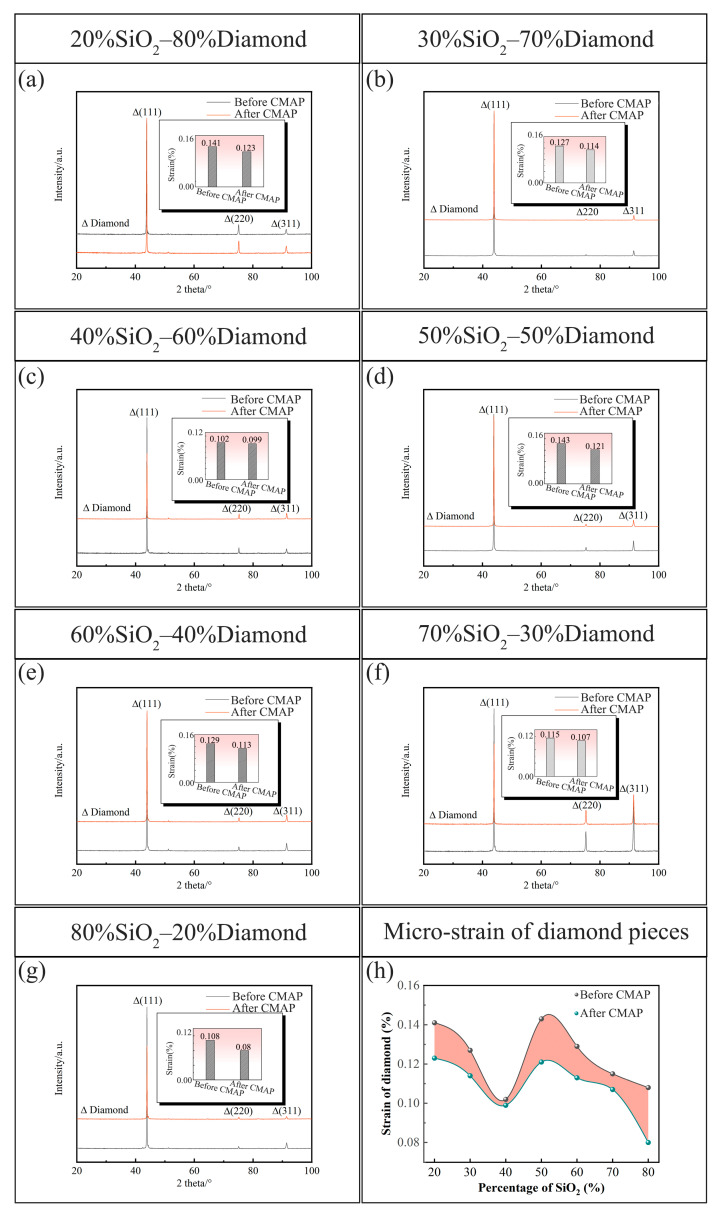
XRD of PCD disks before and after CMAP treatment: (**a**) 20%SiO_2_–80%diamond; (**b**) 30%SiO_2_–70%diamond; (**c**) 40%SiO_2_–60%diamond; (**d**) 50%SiO_2_–50%diamond; (**e**) 60%SiO_2_–40%diamond; (**f**) 70%SiO_2_–30%diamond; (**g**) 80%SiO_2_–20%diamond; (**h**) the calculated microstrain in PCD disks before and after the CMAP process.

Figure 6 shows the XRD patterns and the corresponding calculated microstrain values of the PCD disks before and after the CMAP process. XRD detection was conducted on the central region of each PCD disk to ensure consistent measurement conditions. The Williamson–Hall analysis method was used for the calculation of microstrain in the PCD disks, which is based on the relation between different diffraction angles and the width change in Bragg diffraction caused by the microstrain and grain size effects [17]. The value of the microstrain correlates with the response to crystal defects associated with grain boundary and twin crystals and quantifies the local deviation of the atoms from the ideal position [18,19]. As illustrated in Figure 6a–g, the XRD patterns maintained peaks consistent with the as-deposited PCD wafer that correspond to the (111), (110), and (311) crystal planes of diamond. As illustrated by the inset image in Figure 6a, the microstrain reduction in the PCD disks after CMAP with 20 wt% SiO_2_ slurry consisted in a decrease of 0.018%. This strain relief trend persisted across all samples and exhibited reductions of 0.013%, 0.003%, 0.022%, 0.016%, 0.008%, and 0.028% for SiO_2_ ranging from 30 wt% to 80 wt%, respectively. These variations in microstrain reduction are quantitatively represented in the graphical visualization presented in Figure 6h. In alignment with the findings reported by S. Kaboli [20], the observed reduction in microstrain potentially indicates an atomic recombination on the PCD disk surfaces after CMAP treatment.

**Figure 7:**
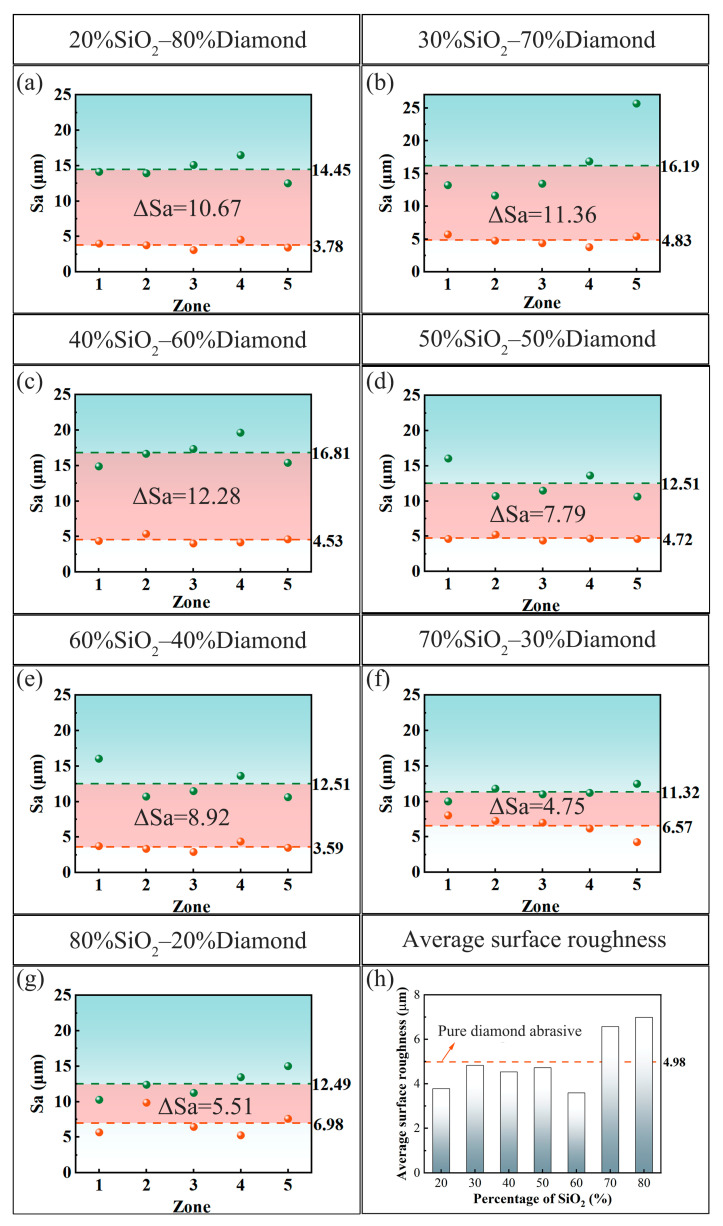
Surface roughness of PCD disks before and after CMAP: (**a**) 20%SiO_2_–80%diamond; (**b**) 30%SiO_2_–70%diamond; (**c**) 40%SiO_2_–60%diamond; (**d**) 50%SiO_2_–50%diamond; (**e**) 60%SiO_2_–40%diamond; (**f**) 70%SiO_2_–30%diamond; (**g**) 80%SiO_2_–20%diamond; (**h**) average surface roughness of PCD after CMAP process.

Figure 7 shows the surface roughness of the PCD disks before and after the CMAP process. In Figure 7a–g, the green dotted lines delineate the average surface roughness prior to the CMAP treatment, while the green balls represent the corresponding five independent measurements. The post-CMAP surface roughness measured results are similarly illustrated by the red dotted line. It was found that the surface roughness of the CMAP-treated PCD disks was in a certain range (3.59–6.98 μm), depending on the different abrasive particles’ contents in the slurries. The surface roughness analysis revealed substantial reductions across all samples, with measured decreases in Sa of 10.67 µm, 11.36 µm, 12.28 µm, 7.79 µm, 8.92 µm, 4.75 µm, and 5.51 µm, as shown in Figure 7a–g, respectively. Figure 7h presents the statistical surface roughness data for the CMAP-treated PCD disks and reveals that the samples processed with slurries with SiO_2_ contents ranging from 20 wt% to 60 wt% demonstrated lower roughness values compared to the sample treated with pure diamond particles. The optimal surface roughness value of 3.59 μm was achieved at the SiO_2_ concentration of 60 wt%. The surface roughness exhibited distinct higher values at the SiO_2_ contents of 70 wt% and 80 wt% because of the lower amounts of abrasive diamond particles. Further, lower levels of diamond particles induced larger contact stress, thereby resulting in more serious surface damage and higher surface roughness.

## 4. Discussion

Bubbles were observed around the carrier during the CMAP process and might have exerted a positive influence on the abrasive polishing of PCD. The effects of mechanical action, bubble cavitation, and chemical action are discussed and analyzed.

### 4.1. Effect of the Mechanical Action

Mechanical abrasion is undoubtedly the predominant mechanism of material removal from PCD disks by diamond particles. Considering the anisotropic nature of different crystal orientations in diamond, single diamond particles in this study were brazed onto 42CrMo steel and ground by a diamond wheel to investigate the wear performance of the different crystal planes under mechanical pressure.

**Figure 8:**
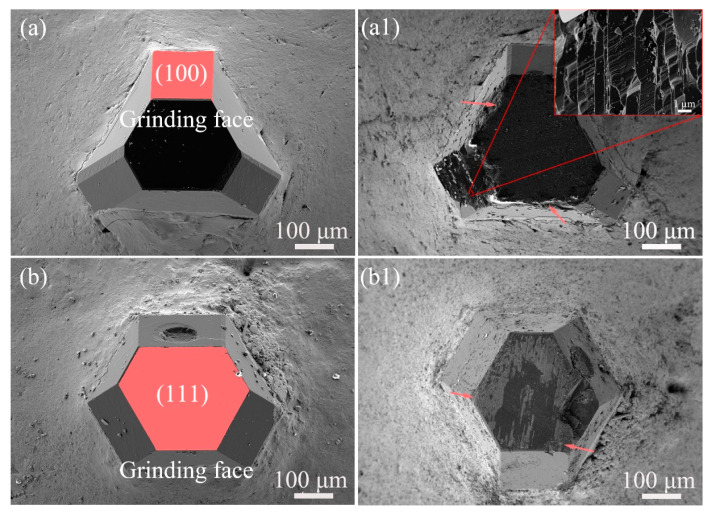
SEM images of single diamond particles brazed on 42CrMo steel: (**a**) before and (**a1**) after grinding on the (100) crystal plane; (**b**) before and (**b1**) after grinding on the (111) crystal plane.

Figure 8 presents the surface morphology of the brazed diamond particles before and after grinding. In Figure 8a,b, it can be observed that the upper part of the diamond particle is exposed, and the lower part demonstrates secure bonding on 42CrMo through the Ni-based solder, ensuring effective mechanical constraint during grinding [21]. The distinct diamond particle edges indicate no apparent graphitization. The (100) and (111) planes marked in pink color were ground for a short time of about ~15 s, and the corresponding morphology is shown in Figure 8(a1,b1) with the edge fractures pointed out by pink arrows. The stress concentration at the edge of diamond will cause the fractures first. In Figure 8(a1) and inset image, massive fractures and a clear stepped cleavage named “river pattern” can be observed [22]. As shown in Figure 8(b1), the ground (111) plane surface exhibited slight wear, but the right edge was broken, with a detachment of the PCD block because of the easy tendency to split of the (111) plane [23]. Considering the large amount of exposed (100) crystal planes on the initially grown surface and the crystal orientation, this also can explain why the CMAP-treated surface exhibited a combination of brittle fracture features, stepped cleavages, and detachment pits. The worn and serious surface cleavage could increase the MRR due to the different contact types [24].

**Figure 9:**
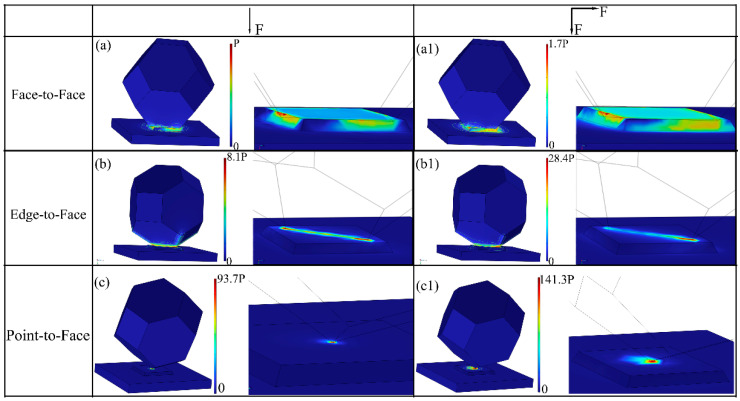
Simulation of stress distribution under different contact types: (**a**,**a1**) face-to-face contact, (**b**,**b1**) edge-to-face contact, and (**c**,**c1**) point-to-face contact.

To further investigate the stress variation caused by different contact types between diamond particles and PCD disk, the stress distribution was simulated using COMSOL 6.2, and the simulation results are shown in Figure 9. The main contact types can be simplified as face-to-face, edge-to-face, point-to-face contacts. The lower part represents a PCD disk with an exposed (100) plane-oriented single grain according to the real surface morphology, and the upper part is assumed to be a diamond abrasive particle (size of 270 µm). Figure 9a shows the simulation results for the stress distribution under face-to-face contact, with a normal force F applied on the diamond particle. It can be observed that the maximum stress appears at the contact edge. The maximum stress on the PCD disk is termed P to intuitively compare the differences in maximum stress on the PCD disk in the presence of different contact types. To account for the condition with a uniform pressure, an external lateral force was applied, and the corresponding results are presented in Figure 9(a1), revealing a maximum stress value of 1.7P. Figure 9(b,b1) show the stress distribution under edge-to-face contact. It can be noted that the maximum stress appears at the contact edge and generates a pressure of 28.4P under the coaction of normal force and lateral force, as shown in Figure 9(b1). In the case of point-to-face contact, the pressure at the contact point reaches 141.3P, as depicted in Figure 9(c1). As illustrated by the stress contour in the figures, the presence of a lateral force not only increases the maximum pressure but also induces radial stress on the contact surface, thereby promoting the initiation and propagation of cracks [23]. In fact, the contact type between the PCD disk and the diamond particles changed constantly due to rotation during the CMAP process, and the edge-to-face contact appeared as the preferred and dominant contact. This contact type could not only achieve a higher grinding efficiency but also produce a lighter surface damage.

**Figure 10:**
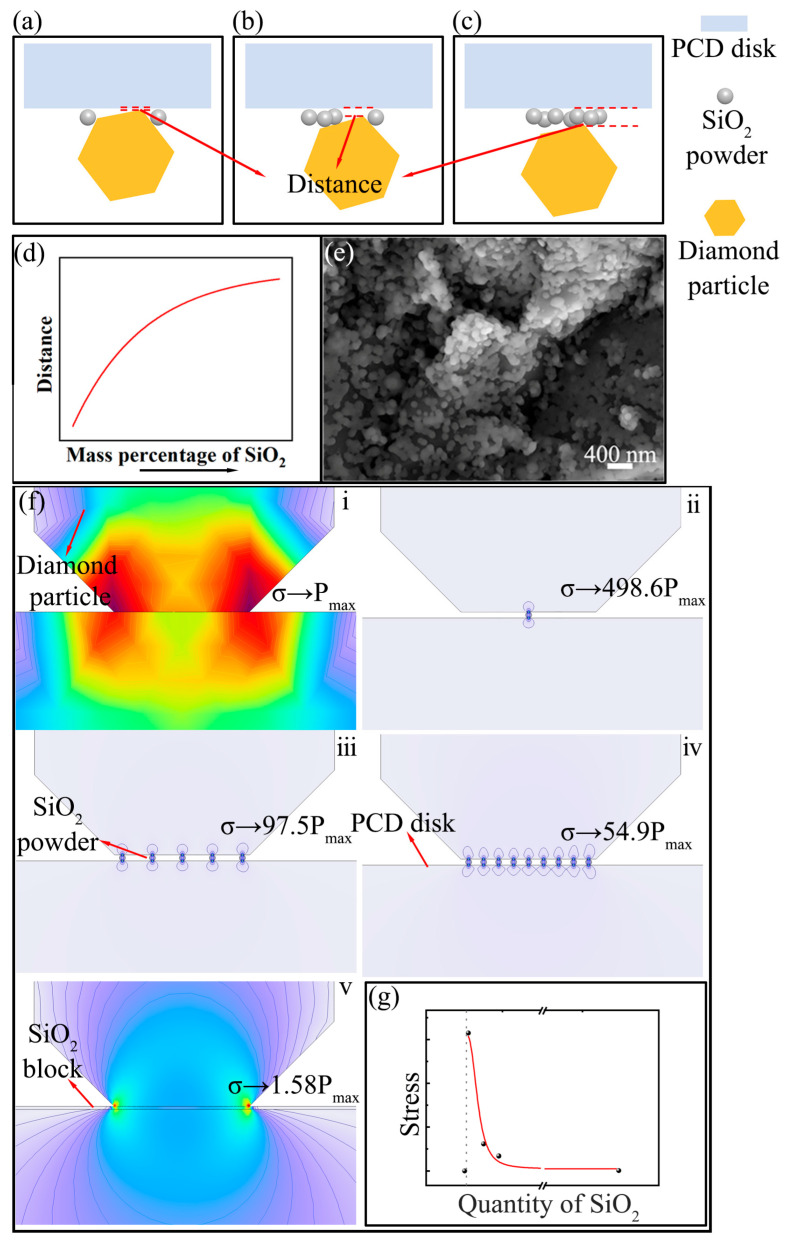
Influence of the changes in mass percentage of the SiO_2_ powder: (**a**–**d**) schematic diagram illustrating the contact between diamond particles and PCD disk; (**e**) morphology of the CMAP-treated PCD; (**f**,**g**) 2D contact stress simulation results under different conditions: (**i**) direct contact between diamond particle and PCD disk; (**ii**–**iv**) increased SiO_2_ particles located between diamond particle and PCD disk; (**v**) SiO_2_ block located between diamond particle and PCD disk.

In Figure 4, it can be observed that the area of the large pits on the PCD disk exhibits a strong correlation with the mass percentages of the SiO_2_ powder, which is governed by the variation in loading pressure. In Figure 1c–f, the thickness of the adsorbed SiO_2_ powder on the diamond particles increases as the mass percentage of SiO_2_ powders rises, and it even exhibits a multilayer structure. Figure 10 presents a schematic diagram depicting both the altered distance between the PCD disk and the diamond particles and the corresponding pressure variation. Figure 10a–d demonstrate that as the mass percentage of SiO_2_ powders increased, the distance between the diamond particles and the PCD disk tended to increase. As depicted in Figure 10a, the adhering SiO_2_ layer was thin, and part of the diamond particles was not covered when SiO_2_ was present in a low mass percentage, which resulted in severe surface damage due to the direct contact between the diamond particles and the PCD disk. In the case of an appropriate ratio between the SiO_2_ powder and diamond particles, the SiO_2_ powder between the PCD disk and the diamond particles acted as solid lubricating layer, which protected the PCD disk from severe damage, as presented in Figure 4(d1, e1). However, when the mass content of SiO_2_ remained at a high level, the correspondingly reduced diamond particle number resulted in a significant increase in contact stress, despite the presence of the SiO_2_ lubricating layer. Such elevated contact stress was capable of inflicting severe damage upon the PCD disk. Figure 10e shows the surface morphology of the CMAP-treated PCD disk in the presence of a large amount of adsorbed SiO_2_ powder that could mitigate surface damage by decreasing the direct contact between the PCD disk and the diamond particles.

Figure 10f presents the 2D stress simulation for an increasing quantity of SiO_2_ based on a simplified geometric model to represent the stress variation trend. In this model, the upper, middle, and lower parts were, respectively, assumed to be a diamond particle, the SiO_2_ powder, and the PCD disk. The stress generated from direct contact between the diamond particles and the PCD disk was assumed to be P_max_. Figure 10g shows the maximum stress analysis results in the graphical form of a curve, revealing a rapid stress reduction trend with an increasing SiO_2_ quantity. Notably, the corresponding stress values consistently remained at magnitudes higher than P_max_. This phenomenon can be attributed to the higher elastic potential energy of the SiO_2_ power, which was transferred to the PCD disk and exerted a positive influence on the removal of diamond material. Based on research by Y. G. Gogotsi [25], diamond undergoes a transformation to graphitization due to the substantial tip stress. In other words, the stress exerted on the PCD disk by the contact force can facilitate the removal of diamond by promoting its graphitization.

### 4.2. Effect of Bubble Cavitation

**Figure 11:**
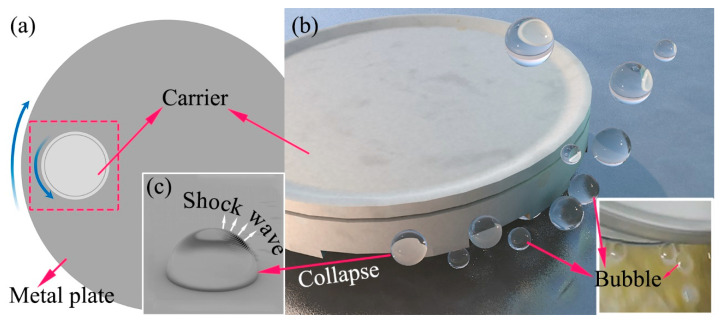
Schematic diagram of the bubbles produced during the CMAP process: (**a**) top view of the metal plate; (**b**) bubbles besides the diamond disk carrier and optical photograph of the actual optical bubbles; (**c**) shock wave while bubble collapse.

Figure 11 depicts a schematic diagram of the bubbles generated during the CMAP process. In Figure 11a, it can be observed that the metal plate rotated clockwise, while the carrier of PCD disks rotated in the opposite direction. The spontaneous decomposition of highly concentrated H_2_O_2_ combined with this relative motion contributed to the formation of bubbles, which could be observed beside the PCD disk carrier, as shown in the inset image at the bottom right of Figure 11b. Figure 11b visually depicts the bubbles located both below and adjacent to the PCD disk carrier on one side.

In general, a shock wave is released when bubbles collapse, as simply shown in Figure 11c, and may affect the removal of PCD materials. This phenomenon has been ignored so far. Based on research by H. Reese [26], the bubbles first inflate and then collapse inward after dozens or hundreds of μs, raising the pressure up to tens of MPa. Moreover, the shock wave generated by the bubbles’ collapse can produce a liquid jet directed towards the PCD disk. When the liquid jet disperses into numerous shear flows directed radially outward along the PCD disk, it exerts a shear stress. Both the liquid jet and the compression wave stimulate a complex wave system in the PCD body consisting of longitudinal, transversal, and Rayleigh waves, possibly causing a slight plastic deformation on the PCD disk surface [27]. In particular, a higher impulse stress is released when two or more bubbles come into contact [28], which will accelerate the physical or mechanical damage to the diamond surface. Furthermore, at the moment of bubble collapse, the inner pressure and temperature will reach large values [29], which accelerates the chemical action on the PCD disk surface.

**Figure 12:**
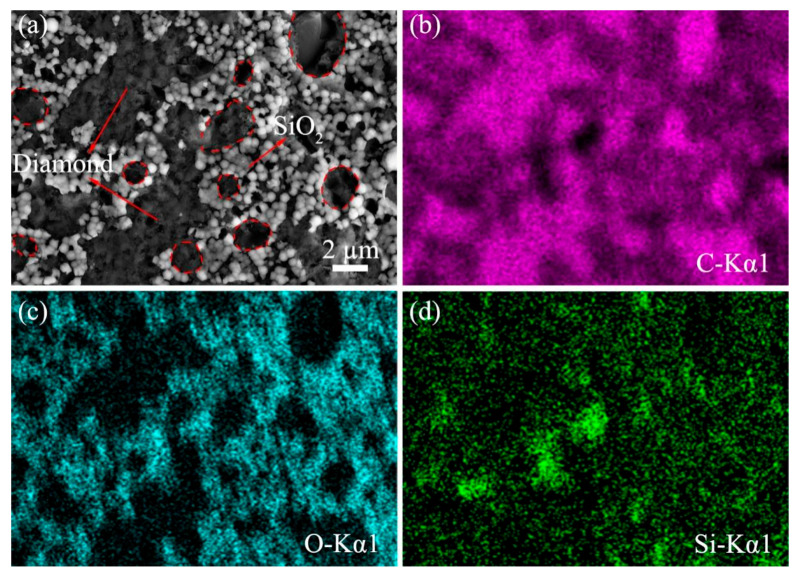
Surface morphology of CMAP-treated samples and element distribution on PCD disk: (**a**) SEM image; (**b**) EDS map of C; (**c**) EDS map of O; (**d**) EDS map of Si.

The CMAP-treated PCD disk was cleaned by ultrasound in ethanol for 15 min, but some special regions on the surface could be observed. The corresponding SEM image and EDS elemental maps are shown in Figure 12. In Figure 12a, it can be noted that spherically gray particles are scattered across the PCD disk. Based on the EDS results shown in Figure 12b–d, O and Si were identified as the main elements in this region. Consequently, these particles were deduced to be SiO_2_ powder. Moreover, the presence of distinct oval zones lacking SiO_2_ particles in the gray region, marked by red-dotted lines, shows the imprints after bubble collapse.

### 4.3. Effect of the Chemical Action

**Figure 13:**
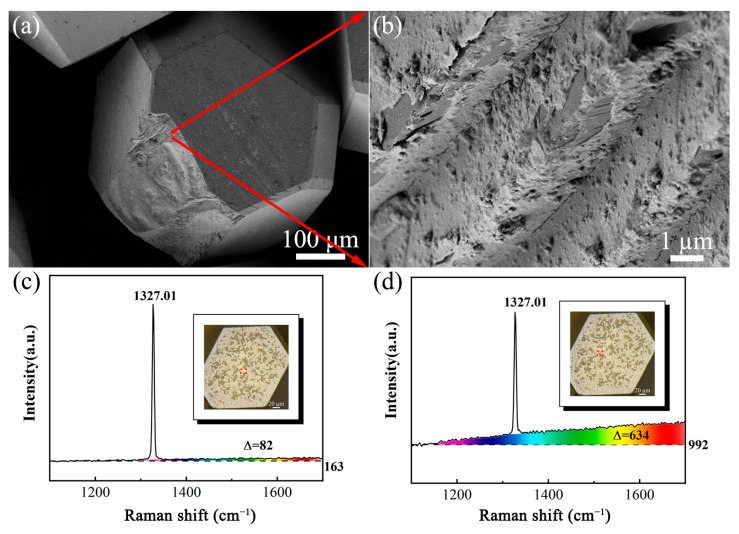
SEM morphology of (**a**) CMAP-treated diamond particle and (**b**) enlarged cleavage fracture area; Raman spectroscopy results for the (**c**) yellow region and (**d**) dark region of the diamond particle.

Figure 13 shows the SEM image of the morphology of a diamond particle and its Raman spectrum after the CMAP process. Figure 13a shows the broken diamond particle, which is scattered with grey-white areas on the surface. An enlarged image of the cleavage region is presented in Figure 13b, where a surficial layer different from the underlying diamond can be observed. EDS characterization was performed for further analysis, but only the C element could be detected (result not presented here). Consequently, Raman spectroscopic analysis was carried out, and the corresponding spectral data are presented in Figure 13c,d. Figure 13c presents the Raman results obtained from the originally yellow region, in which only one peak at 1327.01 cm^−1^ corresponding to diamond was found. Figure 13d shows the Raman results for the dark plaques similar to the grey-white areas depicted in Figure 13a. The diamond peak was detected in the same position. However, the spectral data revealed that the baseline slope in Figure 13d was significantly higher compared to that in Figure 13c, which indicated an increase in amorphous carbon [30,31]. Based on the Raman results, it can be concluded that a transformation from the diamond phase to non-diamond phases occurred on the surface of the diamond particle, which manifested as a delaminated surface layer.

**Figure 14:**
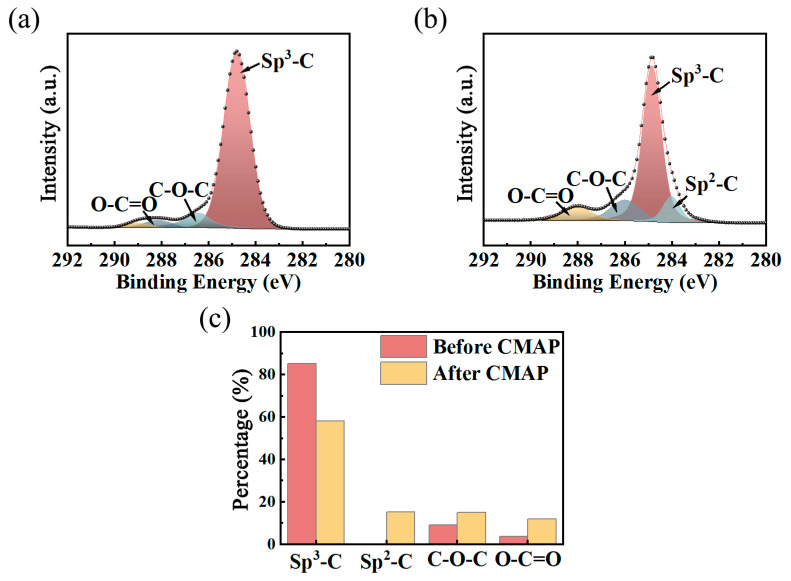
XPS spectra of the PCD disk. (**a**) C1s spectrum before CMAP; (**b**) C1s spectrum after CMAP; (**c**) percentages of the different bonds on the surface.

XPS spectroscopy was conducted to explore the alterations in chemical bonds on the original PCD disk before and after the CMAP process (slurry consisting of 60 wt%SiO_2_ powder and 40 wt% diamond particles). Figure 14a shows the high-resolution spectrum of C1s; the three fitted peaks, each in different colors, represent three chemical bonds: sp^3^-C (284.8 eV), C-O-C (286.3 eV), and O-C=O (288.1 eV). The surface O element may be attributed to the deposited O atoms during PCD growth and the reaction with atmospheric oxygen. The XPS spectrum of the CMAP-treated PCD disk, presented in Figure 14b, revealed an additional characteristic peak at 284 eV, which corresponds to sp^2^-C. The change indicates the partial transformation from sp^3^-C to sp^2^-C during the CMAP process. This phenomenon aligns with the findings of J.W. Ager [32], N. Yang [33], W.J. Zong [34,35], and L. Pastewka [36]. These researchers suggested that the diamond structure with ordered sp^3^-C can be partially transformed into an amorphous sp^3^-C hybridized structure, a disordered sp^2^-C structure, and even sp^0^-C and sp^1^-C structures, in which the amorphous sp^3^-C and sp^2^-C are dominant phases. Figure 14c shows the percentage of different chemical bonds on the PCD disk before and after the CMAP process. It can be observed that the number of C-O-C and O-C=O bonds increased, which can be ascribed to the highly oxidizing ·OH groups existing in the high-concentration H_2_O_2_ solution used, which led to the chemical actions and changes in surface chemical bonds in the PCD.

According to the study of Z. Yuan [37], the C atoms in diamond show a high tendency for a spontaneous reaction with O_2_. This corresponding reaction equation and Gibbs free energy are as follows:

C (s, diamond) + O_2_ (g) → CO_2_ (g), ΔG^θ^ = −397.258 kJ/mol

In addition, the reaction equation between diamond and H_2_O_2_ can be expressed as follows:

C (s, diamond) + 2H_2_O_2_ (l) → CO_2_ (g) + 2H_2_O (g)

And the Gibbs free energy at 298 K can be calculated as follows:

$$\begin{array}{c} {\Delta G_{298}^{\theta }=\Delta H_{298}^{\theta }-298\Delta S}_{298}^{\theta }\\ \;\;\;\;\;\;\;\;=\left[∆H_{298}^{\theta }\left(CO_{2}\right)+2∆H_{298}^{\theta }\left(H_{2}O\right)\right]\\ \;\;\;\;\;\;\;\;\;\;\;\;-\left[∆H_{298}^{\theta }\left(C\left(diamond\right)\right)+2∆H_{298}^{\theta }\left({H_{2}O}_{2}\right)\right]\\ \;\;\;\;\;\;\;\;\;\;-298\{\left[S_{298}^{\theta }\left(CO_{2}\right)+2S_{298}^{\theta }\left(H_{2}O\right)\right]\\ \;\;\;\;\;\;\;\;-{[S}_{298}^{\theta }\left(diamond\right)+S_{298}^{\theta }\left({H_{2}O}_{2}\right)\} \end{array}$$

where $\Delta H_{298}^{\theta }$ and ${\Delta S}_{298}^{\theta }$ are −503.48 kJ/mol and 0.13198 kJ/mol and were calculated based on data from Reference [38]. ${\Delta G}_{298}^{\theta }$ was calculated to be −542.63 kJ/mol, which is even lower than the value obtained for the reaction with O_2_. This indicates a higher tendency for a spontaneous reaction between diamond and H_2_O_2_. In addition, both bubble cavitation and the decomposition of H_2_O_2_ release heat, which promotes the above reactions.

The XPS results revealed changes in the bonds on the PCD disk surface. Based on current studies, variations in C-C bonds on the diamond surface occur as follows: C-C bonds are chemically activated first under mechanical pressure, then deform or even break if the stored strain energy exceeds their bonding energy [39]. The break of C-C bonds usually occurs in the long bonds of the diamond structure due to their lower strength [40]. Next, the broken C-C bond (610 kJ/mol) on the diamond surface may transform into the stronger C-O bond (1077 kJ/mol), C=O bond (799 kJ/mol), or Si-C bonds [8,41]. Finally, part of the C atoms on the diamond surface can be removed in the form of CO, CO_2_, or C chains [42,43].

### 4.4. Cooperative Interaction Between Mechanical Action, Bubble Cavitation, and Chemical Action

**Figure 15:**
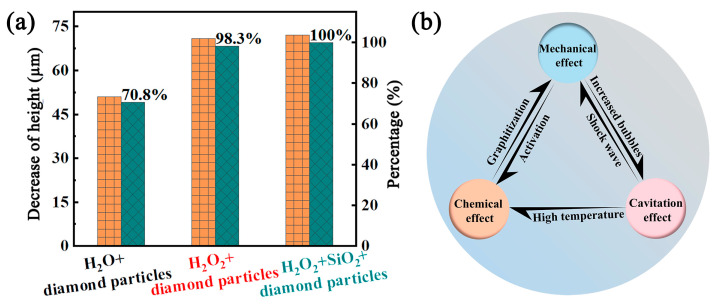
(**a**) Decreased height values after CMAP by different mechanisms; (**b**) schematic diagram of the three cooperative mechanisms in the CMAP process.

The analysis mentioned above indicates the coexistence of multiple synergistic mechanisms during the CMAP process, and each mechanism is helpful for the removal of PCD material. Figure 15 was constructed to provide a comprehensive understanding of the interactions between different mechanisms and their effects on the removal of diamond through several additional designed experiments. Initially, pure diamond particles were used in the additional CMAP processes in both H_2_O and H_2_O_2_ environments, separately, to evaluate and compare the effects of pure mechanical action and mechanical action + bubble cavitation + chemical reaction. Subsequently, to examine the influence of the SiO_2_ powder, a PCD disk was treated with the optimized slurry consisting of 40 wt% diamond particles and 60 wt% SiO_2_ powder in H_2_O_2_ environment. The corresponding height decreases were measured and comparatively evaluated as shown in Figure 15a. The height-decreased values after treatment with pure diamond particles in H_2_O and H_2_O_2_ were 51.1 μm and 71.0 μm, respectively. The latter value represents a 38% improvement, highlighting the significant role of bubble cavitation and chemical reactions in the removal of diamond material. For the case of treatment involving a combination of SiO_2_ and diamond particles, the height reduction value was 72.2 μm, exhibiting an improvement of 1.7% compared with the result obtained with pure diamond particles in H_2_O_2_ environment. That is, even with the optimal CMAP parameters used in this study, the addition of SiO_2_ powder had a limited effect on the removal of diamond material. To quantify the contribution of different mechanisms in PCD removal, based on the experimental results, the height decreases generated in H_2_O and H_2_O_2_ environment were compared with that obtained with the optimal slurry (40 wt% diamond particles and 60 wt% SiO_2_ powder), separately. The result showed that mechanical action played a dominant role in the CMAP process, accounting for 70.8% of the diamond material removal. In contrast, the combination of mechanical action, chemical action, and bubble cavitation contributed by 98.3% to the overall material removal, exhibiting an improvement of 27.5% compared to the contribution of the pure mechanical action. The addition of SiO_2_ in the appropriate proportion had a minimal impact on diamond material removal, contributing only by 1.7%. However, the incorporation of SiO_2_ can change the contact types between the diamond particles and the PCD disk, forming a lubricating layer that mitigates surface damage caused by their direct contact.

The three mechanisms exhibited a cooperative interaction rather than being independent. Figure 15b shows the schematic diagram of the three cooperative mechanisms in the CMAP process. Mechanical action exhibited bidirectional synergistic effects with both chemical reactions and bubble cavitation, facilitating the changes in the diamond C-C bonds and increasing the number of cavitation bubbles. The collapse of more bubbles generated stronger shock waves and higher shear stress, thereby enhancing the mechanical effect. Furthermore, the elevated temperatures produced during bubble collapse not only facilitated the decomposition of H_2_O_2_ but also accelerated the chemical reactions on the PCD disk surface. Consequently, under enhanced chemical action and mechanical pressure, the PCD material could be more easily removed due to the destruction and transformation of the diamond structure. The significant 27.5% improvement in material removal derived from chemical action and bubble cavitation highlights the critical role of the high-concentration H_2_O_2_ environment.

Compared to previous studies, the MRR through the CMAP process was higher. For instance, the maximum MRR (after calculation) was 127.8 µm/(MPa·h) using a (Cu-Sn + Al_2_O_3_) + Fe self-designed wheel at the speed of 500 r/min [44]. In addition, by using a Ti-containing vitrified wheel at the speed of 700 r/min, the calculated MRR for CVD diamond after CMP treatment was 182.1 µm/(MPa·h), and the final surface roughness Ra was about 2.5 µm [45]. In this study, the highest value of MRR was much higher than that mentioned above. Nevertheless, the surface roughness Sa was 3.59 µm, higher than the value of Ra, which may be attributed to the larger measured area in this study. As shown by the comparisons and the experimental results, the optimized slurry could not only improve the MRR but also reduce the surface roughness.

Despite demonstrating significant advantages for PCD pretreatment, the optimized CMAP process presents limitations requiring attention. For instance, the treatment of larger PCD wafers requires the optimization of the final surface uniformity, slurry delivery, and pressure distribution. In addition, the effectiveness may change with the properties of PCD (grain size, crystal plane, and crystal quality). Nevertheless, as for the CMAP process, the higher MRR and reduced surface roughness may offer substantial industrial advantages by decreasing the processing time for large and thick PCD wafers.

## 5. Conclusions

This study conducted a systematic investigation into the chemomechanical abrasive polishing (CMAP) of self-deposited polycrystalline diamond (PCD) wafers utilizing SiO_2_–diamond slurries with varying mass percentages of their components in a 30% H_2_O_2_ solution. The surface morphology, material removal rate (MRR), microstrain, and surface roughness were systematically examined, and the different mechanisms involved in the CMAP process were thoroughly analyzed and discussed. Based on the experimental results and comprehensive analyses, conclusions can be drawn as follows:

(1) The optimal CMAP performance was achieved with a slurry containing 60 wt% SiO_2_ and 40 wt% diamond particles, realizing the MRR of 1039.78 μm/(MPa·h) and the minimum surface roughness (Sa) of 3.59 μm. The new slurry can not only improve the MRR but also reduce the processing costs. Further, the reduced surface roughness can be attributed to the formation of a lubricating layer by the SiO_2_ particles, which effectively mitigated the direct mechanical damage. In addition, a slight reduction in microstrain (0.003–0.028%) was observed, indicating atomic recombination and strain relief on the polished surface.
(2) The synergistic effects of mechanical abrasion, chemical oxidation, and bubble cavitation significantly improved the polishing efficiency. The mechanical action played a predominant role in the diamond material removal, accounting for 70.8% of the total process. This dominance can be attributed to the edge-to-face contact between the diamond particles and the PCD surface, which generated localized high stress and facilitated brittle fracture and grain detachment. Bubble cavitation and chemical reactions collectively contributed by 27.5% to the material removal. The shock waves generated by bubble collapse enhanced the surface shear stress, while chemical action promoted the transformation of sp^3^ carbon to sp^2^ carbon. The incorporated SiO_2_ mainly acted as a lubricating layer and reduced the surface roughness value of 1.39 µm, which contributed by 1.7% to the material removal.

This optimized slurry formulation allows for another application of the CMP technique, establishing a new and efficient method to deal with PCD. Further, it may offer insights for designing multi-mechanism processes for other challenging materials. From an industrial perspective, the 15.5% MRR increase over the MRR obtained using pure diamond translates directly into reduced processing time and cost.

## Figures and Tables

**Table 1:** Parameters for PCD deposition.

Parameter	Value
Power (kW)	20
Pressure (kPa)	12
Substrate temperature (°C)	950 ± 10
CH_4_ concentration (%)	3

## Data Availability

The original contributions presented in this study are included in the article material. Further inquiries can be directed to the corresponding authors.

## References

[1] Zhao G., Li Z., Hu M., Li L., He N., Jamil M. (2019). Fabrication and performance of CVD diamond cutting tool in micro milling of oxygen-free copper. Diam. Relat. Mater..

[2] Yang Q., Zhao J., Huang Y., Zhu X., Fu W., Li C., Miao J. (2019). A diamond made microchannel heat sink for high-density heat flux dissipation. Appl. Therm. Eng..

[3] Ukraintsev E., Kromka A., Janssen W., Haenen K., Takeuchi D., Bábor P., Rezek B. (2021). Electron emission from H-terminated diamond enhanced by polypyrrole grafting. Carbon.

[4] Xiao C., Hsia F.-C., Sutton-Cook A., Weber B., Franklin S. (2022). Polishing of polycrystalline diamond using synergies between chemical and mechanical inputs: A review of mechanisms and processes. Carbon.

[5] Thornton A.G., Wilks J. (1974). The polishing of diamonds in the presence of oxidizing agents. Diam. Res..

[6] Tsai H., Ting C., Chou C. (2007). Evaluation research of polishing methods for large area diamond films produced by chemical vapor deposition. Diam. Relat. Mater..

[7] Thomas E.L.H., Mandal S., Brousseau E.B., A Williams O. (2014). Silica based polishing of {100} and {111} single crystal diamond. Sci. Technol. Adv. Mat..

[8] Werrell J.M., Mandal S., Thomas E.L.H., Brousseau E.B., Lewis R., Borri P., Davies P.R., Williams O.A. (2017). Effect of slurry composition on the chemical mechanical polishing of thin diamond films. Sci. Technol. Adv. Mat..

[9] Kubota A., Nagae S., Touge M. (2016). Improvement of material removal rate of single-crystal diamond by polishing using H_2_O_2_ solution. Diam. Relat. Mater..

[10] Mandal S., Thomas E.L., Gines L., Morgan D., Green J., Brousseau E.B., Williams O.A. (2018). Redox agent enhanced chemical mechanical polishing of thin film diamond. Carbon.

[11] Sun B., Zhang X., Lin Z. (1993). Growth mechanism and the order of appearance of diamond (111) and (100) facets. Phys. Rev. B.

[12] Frenklach M. (1989). The role of hydrogen in vapor deposition of diamond. J. Appl. Phys..

[13] Srikanth V.V.S.S. (2011). Review of advances in diamond thin film synthesis. Proc. Inst. Mech. Eng. Part C J. Mech. Eng. Sci..

[14] Lee S.T., Peng H.Y., Zhou X.T., Wang N., Lee C.S., Bello I., Lifshitz Y. (2000). A Nucleation Site and Mechanism Leading to Epitaxial Growth of Diamond Films. Science.

[15] Ralchenko V., Pleuler E., Lu F., Sovyk D., Bolshakov A., Guo S., Ang W., Ontar I., Khomich A., Zavedeev E. (2012). Fracture strength of optical quality and black polycrystalline CVD diamonds. Diam. Relat. Mater..

[16] Zheng Y., Ye H., Thornton R., Knott T., Ochalski T.J., Wang J., Liu J., Wei J., Chen L., Cumont A. (2020). Subsurface cleavage of diamond after high-speed three-dimensional dynamic friction polishing. Diam. Relat. Mater..

[17] Williamson G.K., Hall W.H. (1953). X-ray line broadening from filed aluminium and wolfram. Acta Metall..

[18] Chattot R., Le Bacq O., Beermann V., Kühl S., Herranz J., Henning S., Kühn L., Asset T., Guétaz L., Renou G. (2018). Surface distortion as a unifying concept and descriptor in oxygen reduction reaction electrocatalysis. Nat. Mater..

[19] Xia Z., Guo S. (2019). Strain engineering of metal-based nanomaterials for energy electrocatalysis. Chem. Soc. Rev..

[20] Kaboli S., Burnley P.C. (2018). Direct observations of crystal defects in polycrystalline diamond. Mater. Charact..

[21] Zheng X., Zheng K., Chang J., Qu S., Jia W., Li Z., Yu S., Gao J., Ma Y. (2022). Microstructure, mechanical properties and reciprocating wear properties of diamond grits-reinforced NiCrBSi composite coatings on 42CrMo. Surf. Coat. Technol..

[22] Wu H., Huang H., Jiang F., Xu X. (2016). Mechanical wear of different crystallographic orientations for single abrasive diamond scratching on Ta12W. Int. J. Refract. Met. Hard Mater..

[23] Wang K., Zhang J., Kang J., Zhang H. (2023). Analysis of diamond wear morphology and segment wear evolution during the process of hard granite sawing. Int. J. Refract. Met. Hard Mater..

[24] De Pellegrin D.V., Corbin N.D., Baldoni G., Torrance A.A. (2009). Diamond particle shape: Its measurement and influence in abrasive wear. Tribol. Int..

[25] Gogotsi Y.G., Kailer A., Nickel K.G. (1999). Transformation of diamond to graphite. Nature.

[26] Reese H., Ohl S.-W., Ohl C.-D. (2023). Cavitation bubble induced wall shear stress on an elastic boundary. Phys. Fluids.

[27] Abbondanza D., Gallo M., Casciola C.M. (2022). Cavitation over solid surfaces: Microbubble collapse, shock waves, and elastic response. Meccanica.

[28] Naing N.M.T., Park J., Hyun S.-H., Jung R.-T. (2022). Impact loads generated by tandem cavitation bubble on solid wall. J. Hydrodyn..

[29] Brenner M.P., Hilgenfeldt S., Lohse D. (2002). Single-bubble sonoluminescence. Rev. Mod. Phys..

[30] McQuillan A.K., Clements W.R.L., Stoicheff B.P. (1970). Stimulated Raman Emission in Diamond: Spectrum, Gain, and Angular Distribution of Intensity. Phys. Rev. A.

[31] Ager J.W., Drory M.D. (1993). Quantitative measurement of residual biaxial stress by Raman spectroscopy in diamond grown on a Ti alloy by chemical vapor deposition. Phys. Rev. B.

[32] Kawaguchi K., Wang Y., Xu J., Ootani Y., Higuchi Y., Ozawa N., Kubo M. (2021). Atom-by-Atom and Sheet-by-Sheet Chemical Mechanical Polishing of Diamond Assisted by OH Radicals: A Tight-Binding Quantum Chemical Molecular Dynamics Simulation Study. ACS Appl. Mater. Interfaces.

[33] Yang N., Zong W., Li Z., Sun T. (2014). Amorphization anisotropy and the internal of amorphous layer in diamond nanoscale friction. Comp. Mater. Sci..

[34] Zong W., Zhang J., Liu Y., Sun T. (2014). Achieving ultra-hard surface of mechanically polished diamond crystal by thermo-chemical refinement. Appl. Surf. Sci..

[35] Zong W., Cheng X., Zhang J. (2016). Atomistic origins of material removal rate anisotropy in mechanical polishing of diamond crystal. Carbon.

[36] Pastewka L., Moser S., Gumbsch P., Moseler M. (2010). Anisotropic mechanical amorphization drives wear in diamond. Nat. Mater..

[37] Yuan Z., Zheng P., Wen Q., He Y. (2017). Chemical kinetics mechanism for chemical mechanical polishing diamond and its related hard-inert materials. Int. J. Adv. Manuf. Technol..

[38] Barin I., Knacke O., Kubaschewski O. (1977). Thermochemical Properties of Inorganic Substances.

[39] Fairchild B.A., Rubanov S., Lau D.W.M., Robinson M., Suarez-Martinez I., Marks N., Greentree A.D., McCulloch D., Prawer S. (2012). Mechanism for the Amorphisation of Diamond. Adv. Mater..

[40] Peguiron A., Moras G., Walter M., Uetsuka H., Pastewka L., Moseler M. (2016). Activation and mechanochemical breaking of C–C bonds initiate wear of diamond (110) surfaces in contact with silica. Carbon.

[41] Thomas E.L., Nelson G.W., Mandal S., Foord J.S., Williams O.A. (2014). Chemical mechanical polishing of thin film diamond. Carbon.

[42] Shi Z., Jin Z., Guo X., Yuan S., Guo J. (2019). Insights into the atomistic behavior in diamond chemical mechanical polishing with %OH environment using ReaxFF molecular dynamics simulation. Comp. Mater. Sci..

[43] Guo X., Yuan S., Wang X., Jin Z., Kang R. (2019). Atomistic mechanisms of chemical mechanical polishing of diamond (1 0 0) in aqueous H_2_O_2_/pure H_2_O: Molecular dynamics simulations using reactive force field (ReaxFF). Comput. Mater. Sci..

[44] Xu H., Zang J., Tian P., Wang Y., Yu Y., Lu J., Xu X., Zhang P. (2018). Rapid grinding CVD diamond films using corundum grinding wheels containing iron. Int. J. Refract. Met. Hard Mater..

[45] Xu H., Zang J., Tian P., Yuan Y., Wang Y., Yu Y., Lu J., Xu X., Zhang P. (2018). Surface conversion reaction and high efficient grinding of CVD diamond films by chemically mechanical polishing. Ceram. Int..

